# The Role of Radiomic Analysis and Different Machine Learning Models in Prostate Cancer Diagnosis

**DOI:** 10.3390/jimaging11080250

**Published:** 2025-07-23

**Authors:** Eleni Bekou, Ioannis Seimenis, Athanasios Tsochatzis, Karafyllia Tziagkana, Nikolaos Kelekis, Savas Deftereos, Nikolaos Courcoutsakis, Michael I. Koukourakis, Efstratios Karavasilis

**Affiliations:** 1 Medical Physics Laboratory, School of Medicine, Democritus University of Thrace, 68100 Alexandroupolis, Greece; 2 Medical Physics Laboratory, School of Medicine, National and Kapodistrian University of Athens, 11527 Athens, Greece; iseimen@med.uoa.gr; 3 Ygeia Private Hospital, 15123 Athens, Greece; thanasis.tsochatzis@hotmail.com; 4 Department of Radiology, School of Medicine, Democritus University of Thrace, 68100 Alexandroupolis, Greecesdeftereos@med.duth.gr (S.D.); ncourcou@med.duth.gr (N.C.); 5 Research Unit of Radiology and Medical Imaging, 2nd Department of Radiology, Medical School, National and Kapodistrian University of Athens, 11527 Athens, Greece; kelnik@med.uoa.gr; 6 Department of Radiotherapy/Oncology, University Hospital of Alexandroupolis, Democritus University of Thrace, 68100 Alexandroupolis, Greece; mkoukour@med.duth.gr

**Keywords:** machine learning, radiomics, prostate cancer, biparametric magnetic resonance imaging, prostate-specific antigen, prostate cancer diagnosis

## Abstract

Prostate cancer (PCa) is the most common malignancy in men. Precise grading is crucial for the effective treatment approaches of PCa. Machine learning (ML) applied to biparametric Magnetic Resonance Imaging (bpMRI) radiomics holds promise for improving PCa diagnosis and prognosis. This study investigated the efficiency of seven ML models to diagnose the different PCa grades, changing the input variables. Our studied sample comprised 214 men who underwent bpMRI in different imaging centers. Seven ML algorithms were compared using radiomic features extracted from T2-weighted (T2W) and diffusion-weighted (DWI) MRI, with and without the inclusion of Prostate-Specific Antigen (PSA) values. The performance of the models was evaluated using the receiver operating characteristic curve analysis. The models’ performance was strongly dependent on the input parameters. Radiomic features derived from T2WI and DWI, whether used independently or in combination, demonstrated limited clinical utility, with AUC values ranging from 0.703 to 0.807. However, incorporating the PSA index significantly improved the models’ efficiency, regardless of lesion location or degree of malignancy, resulting in AUC values ranging from 0.784 to 1.00. There is evidence that ML methods, in combination with radiomic analysis, can contribute to solving differential diagnostic problems of prostate cancers. Also, optimization of the analysis method is critical, according to the results of our study.

## 1. Introduction

Prostate cancer (PCa) is the second-most common cancer in men worldwide and the fifth leading cause of cancer-related deaths among men [1]. The early detection and grading of PCa play a crucial role in patient management, therapy planning, and long-term survival evaluation. Serum Prostate-Specific Antigen (PSA) and Digital Rectal Examination (DRE) are the most widely used PCa screenings in clinical practice, following the European Association of Urology (EAU)—European Society for Radiotherapy and Oncology (ESTRO)—International Society of Geriatric Oncology (SIOG) Guidelines [2]. The traditional PSA cutoff of 4 ng/mL imposes histopathological verification through biopsy [3,4]. However, the study by Marriel et al. indicates that PSA has a low specificity of 20%, disputing the usefulness of PSA in the accurate diagnosis of clinically significant prostate cancer (cs-PCa), since they recorded many cases with low PSA value and cs-PCa and, contrarily, high PSA value in benign pathologies like prostate hypertrophy [5].

In the studies of Gershmann et al., only approximately 18% of men with elevated PSA were diagnosed with cancer. The remaining 82% of men underwent biopsies without actually having prostate cancer and were exposed to potential complications such as bleeding, infection, and urinary retention [6]. Thus, there is a need to develop an algorithm that, by taking clinical, demographic, and imaging information, will more accurately define the cases that truly need a biopsy [7].

Multiparametric Magnetic Resonance Imaging (mpMRI) can be considered a sophisticated diagnostic approach for the detection, differentiation, and risk classification of PCa since it provides imaging biomarkers from conventional and advanced imaging techniques, such as high-resolution T2-weighted (T2W), diffusion-weighted (DWI) and dynamic contrast-enhanced sequences (DCE) [8,9]. Mp-MRI diagnostic accuracy in PCa further increased the area under the receiver operating characteristic curve (AUC = 0.893) value when expert radiologists followed the Prostate Imaging Reporting and the Data System Version 2 (PI-RADS v2), which is considered the most promising approach for PCa screening with high diagnostic accuracy AUC = 0.893 to PCa differentiation [10].

The lack of expert prostate imaging radiologists and the interobserver variability in the interpretation of mp-MRI, the large spectrum of acquisition parameters, and the heterogeneity of PCa tumors are factors that significantly reduce the sensitivity and specificity of the imaging method [11,12,13].

Consequently, there is a need for objective indices to mitigate radiologists’ faults. Radiomic analysis and machine learning (ML) methods offer an objective approach for evaluating MRI data by extracting imaging features usually not easily detectable by the radiologist’s eye [14,15]. Radiomic analysis allows the mining of quantitative characteristics like texture, size, and shape from clinical images, like MRI, useful to diagnose and differentiate PCa [14]. ML is adept at analyzing vast, complex datasets without prior biomedical hypotheses, uncovering insights that may be clinically relevant. As a result, ML, particularly in the area of classification, is being integrated into radiomic research to refine prostate cancer evaluations and reduce subjectivity [16]. Although ML and radiomics combined are promising diagnosis tools in prostate cancer, they face limitations related to the high susceptibility to variations in acquisition parameters, the sample size, the statistical methodological approach, and the heterogeneous datasets mixing peripheral zone (PZ) with transition zone (TZ) tumors [17].

The main purpose of this study was to evaluate the diagnostic performance of different ML approaches to detect and assess PCa aggressiveness using standardized MRI protocol across many centers. In particular, we investigated the diagnostic performance of ML to differentiate the different PCa grades by (a) applying seven ML models and (b) changing the input variables.

## 2. Materials and Methods

### 2.1. Patient Population

Our sample size consisted of 214 participants with increased PSA or clinical symptoms related to prostate dysfunction who underwent MRI examination in three different imaging centers equipped with four different MRI systems. The data comprised four datasets: dataset 1 (86 exams on a 3T MRI), dataset 2 (21 exams on a 1.5 T MRI), dataset 3 (88 exams on a 3 T MRI), and dataset 4 (19 exams on a 1.5T MRI). All participants were given Τransrectal Ultrasound Guided (TRUS) biopsy in order to validate the lesion type.

Exclusion criteria were (1) prior therapy history for PCa patients, including antihormonal therapy, radiation, cryotherapy, or prostatectomy, (2) incomplete information or (3) severe imaging artifacts of the MRI images, and (4) lack of serum PSA level.

### 2.2. MRI Acquisitions

The image acquisition protocol was harmonized across all centers since the core scientific group had set minimum requirements, such as high-resolution T2W images of at least 3.0 mm gapless slice distance in the axial plane and DWI images with the same slice distance, 2 b values with the high b value at least 1000 s/mm^2^.

### 2.3. MRI Lesion Segmentation

All individual lesions were manually delineated on T2W based on PI-RADSv2.1 reports by an expert radiologist with ten years of experience in examining PCa lesions using ITK-SNAP [18].

### 2.4. Image Pre-Processing

Firstly, we applied Bias correction on T2W and DWI images to compensate for intensity non-uniformities using N4 Bias Correction on SimpleITK Python 2.1.1.2 library [19]. Then, we performed basic normalization by scaling and shifting the values of the whole image to a mean signal value of 300 and a standard deviation of 100 [12]. Finally, a resampling pixel sampling 1 × 1 × 1 mm^3^ with sitkBSpline interpolator and fixed bin-width (FBW) discretization equal to 10 for T2W images and 5 for DWI images were performed to handle differences in image resolution [20,21]. All the pre-processing steps were applied using the open-source software Pyradiomics v1.3.0 [22].

### 2.5. Feature Extraction

Radiomic features were extracted from the pre-processed T2W and DWI images using the Pyradiomics v1.3.0, following the Imaging Biomarkers Standardization Initiative (IBSI) processing protocol [22,23]. In particular, in the extracted texture features were included (i) shape-based features (*n* = 14), (ii) first-order features (*n* = 18), (iii) gray-level co-occurrence matrix (GLCM) (*n* = 22) features, (iv) gray-level size zone matrix (GLSZM) features (*n* = 19), (v) gray-level run length matrix (GLRLM) features (*n* = 14), and (vi) gray-level dependence matrix (GLDM) features (*n* = 14). These features are enabled in the Pyradiomics code by default. Appendix A includes more details about extracted features. Before proceeding to the next steps, the Radiomics Quality Score (RQS) checklist was applied to ensure the methodological quality of the radiomics study and to enhance the generalizability of our model, achieving a score of 70%. (25/36 of total score) [20,24,25].

### 2.6. Feature Selection and Dimension Reduction

All radiomics features were normalized before feature selection, with Z scores standardized to eliminate features’ distortions in the range of values [26]. Radiomic approaches generate many features, leading to a high-dimensional dataset. The high dimensionality diminishes the classifier’s performance. In this study, the Gini index algorithm-based feature is selected [27]. The feature selection and dimension reduction were applied by the Orange Data Mining tool (v.3.36.1) [28].

### 2.7. Model Development

Seven algorithms, K-Nearest Neighbors (k-NN), Naive Bayes (NB), logistic regression (LR), Support Vector Machine (SVM), Decision Tree (DT), Random Forest (RF) and Neural Network (NN), were chosen as classifiers in the classification model analysis of this study. The Orange Data Mining tool (v.3.36.1) was employed in the development of the above machine learning models [28]. The best-performing parameters of classifiers are generally selected for our model development.

The k-NN was set to 6 neighbors per datapoint, Euclidean distance metric with uniform weight distribution [29]. Appendix B justifies the set of k = 6. The NB algorithm was used without any parametric changes. The regularization for LR was set to Least Absolute Shrinkage and Selection Operator (LASSO)-L1 regularization [30]. The SVM type was applied with Cost (C) = 1 and Regression Loss Epsilon (ε) = 0,10, Polynomial Kernel and iteration limit was set to 100, and numerical tolerance to 0.0010. The DT was binary induced with a minimum of 3 instances in leaves, minimum splitting of subsets up to 2 with a maximum tree depth of 200 [31]. The RF had 10 trees with a minimum split of subsets up to 5 [31]. NN contained 3 hidden layers with 100 neurons per hidden layer, 100 maximum number of iterations, and a Rectified Linear Unit (ReLU) activation function [32].

### 2.8. Performance Evaluation

The performance of ML classifiers was evaluated using the AUC on 10 cross-validation. The validation performance of the algorithms was compared by receiver operating characteristic (ROC) curve analysis. These attributes were computed by default in the Orange Data Mining tool (v.3.36.1) for classification models [28].

Additionally, a held-out test set validation was conducted by using datasets 1, 2, and 3 for training, while dataset 4 was reserved as a test set, allowing for a more thorough evaluation of the models’ generalization performance. This was only applied to the model that differentiated benign from malignant prostate lesions regardless of their location, since dataset 2 and 4, which could be used for a test set, included a limited number of cases with a lesion in the PZ.

The DeLong test was applied to compare the performance of classifiers by testing whether the difference between their area under the curve (AUC) values is statistically significant, using Python 3.9 Software [33].

**Figure 1:**
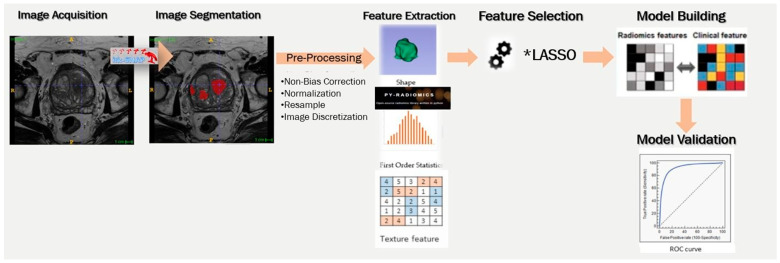
Schematic representation of the pipeline process followed in this study for different machine learning models in prostate cancer diagnosis. *LASSO: Least Absolute Shrinkage and Selection Operator.

A schematic representation of the pipeline process followed in this study is illustrated in Figure 1.

## 3. Results

### 3.1. Clinical Characteristics

One hundred thirteen participant patients (53%) were diagnosed with benign lesions, and the remaining 101 (47%) with malignant lesions. In particular, biopsy showed a Gleason score (GS) ≤ 6 in 113 patients (53%) and a GS > 6 in 101 patients (47%). The international Society of Urological Pathology (ISUP) group was distributed in the low-risk group (ISUP = 1) in 113 (52.80%) patients, in the intermediate-risk group (ISUP = 2 and ISUP = 3) in 83 (38.79%) patients, and in the high-risk group (ISUP > 3) in 18 (8.41%) patients. The most lesions were detected in PZ in 158 (74%) patients, and the other 56 (26%) lesions were located in οther prostate zones. Further details on patient clinical characteristics can be found in Table 1.

### 3.2. Predictive Ability of Differentiation of Benign and Malignant Prostate Lesions

Firstly, the dataset was grouped based on GS of lesion, independent of lesion location. Class A included benign prostate lesions (GS ≤ 6), and Class B malignant prostate lesions (GS > 6). The different AUC results of all the models used for T2W dataset, DWI dataset, and their combination are presented in Table 2.

Table 3 presents the statistical comparison of AUCs between models using the DeLong test to assess the significance of performance differences.

Model performance based on held-out cross-validation is presented in Table 4.

### 3.3. Predictive Ability of Differentiation of Low-Risk Lesions from Intermediate-Risk Lesions on the Peripheral Zone

The dataset was divided based on the ISUP score of lesions that were located in the PZ of the prostate gland. Class A included low-risk lesions with ISUP = 1 (GS = 6), and Class B intermediate-risk lesions with ISUP = 2 (GS = 3 + 4) and ISUP = 3 (GS = 4 + 3). AUC values of various ML models across different datasets are presented in Table 2.

### 3.4. Predictive Ability of Differentiation of ISUP = 2 and ISUP = 3 on the Peripheral Zone

**Figure 2:**
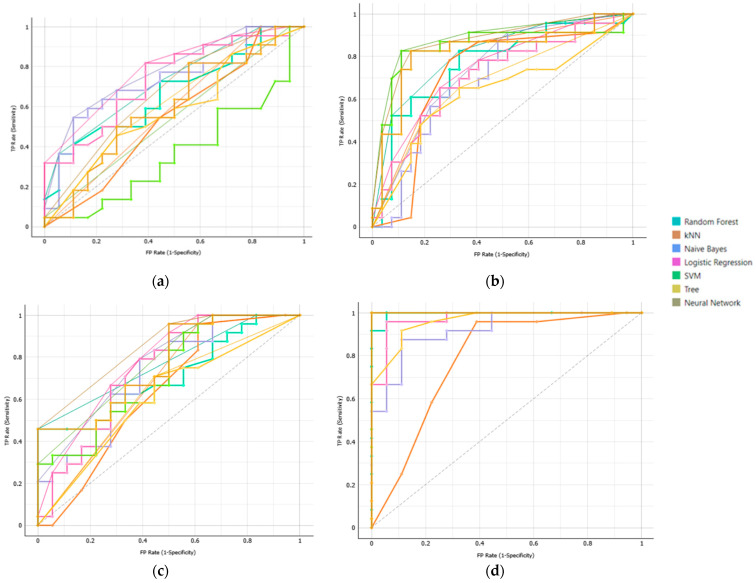
Receiver operating characteristic (ROC) curve analysis to evaluate machine learning algorithms efficiency in classification intermediate-risk lesions with International Society Of Urological Pathology (ISUP) group 2 from ISUP group 3 using (**a**) T2-weighted (T2W) model, (**b**) diffusion-weighted imaging (DWI) model, (**c**) T2W and DWI model, and (**d**) T2WI, DWI, and (Prostate-Specific Antigen) PSA model, respectively. Light Blue Line: Random Forest Algorithm; Brown Line: kNN; Blue Line: Naïve Bayes; Magenta Line: logistic regression; Green Line: Support Vector Machine(SVM); Yellow: Decision Tree; Gray Line: Neural Network.

The dataset was divided into classes based on the ISUP score of lesions located in the peripheral zone (PZ) of the prostate gland. Class A included lesions with ISUP = 2, and Class B lesions with ISUP = 3. AUC results across different datasets and all the models are presented in Table 2, and the corresponding ROC curve is illustrated in Figure 2.

## 4. Discussion

The past decade, the research community has stratified post-processing methods such as radiomic analysis combined with ML models to diagnose clinically significant prostate cancer. In this study, we validated the noteworthy contribution of radiomics, trying to reveal the impact of different methodological approaches in the final model’s efficiency. Specifically, we investigated the effect of the different inputs in ML models and the effect of the applied ML methods in different clinical queries.

Our results have shown that the models’ efficiency is highly dependent on the input variables, as expected. In most of the examined scenarios, the T2W and DWI-weighted-derived radiomics either as independent or combined inputs shown limited clinical usability. The models’ efficiency was significantly improved by introducing PSA clinical index independently of lesion location or the degree of malignancy.

The positive effect of introducing a clinical variable on the model performance is in line with the existing literature. Marvaso et al. created four different models. Model 1, including only clinical variables (PSA, pre-operative GS, ISUP, Tumor Nodule Metastasis (TNM) stage and age), achieved AUC = 0.68. Model 2 combined the aforementioned clinical and radiological features (ADC, PI-RADS, lesion volume) and showed a significant improvement, with an AUC of 0.79. Model 3, which integrated prior clinical data with radiomic features, achieved an AUC of 0.71. Finally, Model 4, which combined all features, achieved the highest AUC of 0.81, indicating that the most accurate predictions of PCa pathology were obtained when all variables were incorporated [34].

Similar results were observed in the study by Dominguez et al., where an LR classifier was used to distinguish clinical insignificant (ciPCa) and csPCa, and its performance was improved notably with the inclusion of both radiological (T2W- and Apparent Diffusion Coefficient (ADC)-derived radiomics, prostate volume) and PSA clinical feature (CL) [35]. Specifically, the individual variables, CL, T2W, ADC showed AUC 0.76, 0.85, and 0.81, respectively, and their combination 0.91 [35].

Controversially, there are studies in which the integration of PSA with radiologically derived quantitative metrics did not contribute to further increasing and in some cases decreasing the model’s rendering. In the study conducted by Gong et al., the addition of PSA to the T2W-DWI yielded restricted improvement in model performance. Specifically, the clinical model achieved AUCs of 0.723, while the T2W-DWI model reached 0.788, and the combined T2W-DWI-clinical model slightly improved to 0.780 [36].

However, Lu et al. compared multiple models for PCa prediction in a validation cohort, where the TZ-PSA density model yielded a relatively low AUC of 0.592. In contrast, radiomic models with the ADC-based radscore reaching 0.779, the T2W-based radscore 0.808, the fusion radscore 0.844, and the radiomic nomogram incorporating TZ volume achieving the highest AUC of 0.872. This discrepancy may be attributed to differences in dataset composition (57.4% of their cases located in ΤΖ) [37].

Moreover, there are numerous studies including only radiological metrics in their models and achieving palatable efficiency. A recent review of Antonil et al. presented 14 studies that introduced only radiological features in computational models to discriminate cs-PCa and ciPCa. In line with our results, efficiency was improved when they were introduced to more than one source of feature (in most cases T2w and DWI). AUC ranged from 0.68 to 0.81 when DWI or T2w imaging data were introduced as individual inputs, while their combination achieved AUC 0.73 to 0.98 [38].

Our AUC values were observed to be lower than those reported by some studies in the literature. We assume that this is because most studies used data from a single MR system and applied higher, more sensitive to lesion detection b-values than ours. For example, Jin et al. used b = 2000 s/mm^2^, Jing et al. b = 1500 s/mm^2^, and Hamm et al. b = 1400 s/mm^2^—all acquired on 3T scanners. A notable exception is Castillo et al., who used data from both 1.5T and 3T systems with b-values ranging from 600 to 1000, reporting an AUC of 0.72, similar to our results [39,40,41,42].

The selection of ML algorithm in PCa classification depends on data characteristics like data dimensionality, feature correlations, and computational resources. Performance evaluation through cross-validation and performance metrics is crucial to determine the most suitable algorithm [43]. The comparison of seven ML algorithms in this survey provides greater reliability for our model.

Classification performance of our ML models in the prediction of csPCa was shown to be improved, incorporating T2W, DWI and PSA. Among models, NN achieved the highest performance (AUC = 0.992), followed by SVM (AUC = 0.957), DT (AUC = 0.953), and RF (AUC = 0.946). The efficiency of these models was consistent across different clinical questions posed, highlighting their robustness and generalizability compared to the traditional ML models LR, kNN, and NB, whose performance varied across different tasks. Specifically, LR and kNN showed moderate performance AUC = 0.884 and 0.868, respectively, whereas NB had the lowest performance (AUC = 0.830).

The models’ generalization performance approved relatively consistently across the various evaluation strategies employed, including cross-validation and a held-out validation set. NN exhibited the highest performance during model development, achieving an AUC of 0.992 in cross-validation. Its performance on the held-out set remained robust (AUC = 0.936), indicating strong generalization during initial validation. Similarly, SVM presented a perfect AUC of 1.000 on the held-out set and high performance of AUC = 0.957 in cross-validation. RF and DT delivered strong results during cross-validation (AUCs of 0.946 and 0.953, respectively). While their performance saw a drop on the held-out set (AUCs of 0.814 and 0.929), they still exhibited solid generalization. In contrast, LR, kNN, and NB presented moderate performance during model development (AUC_cross_validation, kNN:0.868; NB: 0.830 and LR: 0.884). However, they maintained consistent performance across datasets (AUC_held_out, kNN: 0.764; NB: 0.700; LR: 0.764) These findings indicate the high predictive abilities of deep learning models compared with traditional ML models, which have less capture ability to detect complex feature interactions.

According to Nematollahi et al. and other related studies, the performance of various supervised ML models using mpMRI or bpMRI data for prostate cancer (PCa) diagnosis varies considerably [31]. Across the published studies different methodological approaches were observed, as regards to the input variables, data sample, and pre- and post-processing analysis steps. Therefore, logistic regression (LR) consistently demonstrates strong performance, with reported AUCs ranging from 0.82 to 0.97 [31]. SVM also perform well, with AUCs between 0.727 and 0.89 for mpMRI and up to 0.85 for bpMRI [38,44,45]. kNN achieves AUCs of 0.82–0.88 (mpMRI) and up to 0.84 (bpMRI), while RF shows AUCs ranging from 0.76 to 0.94 [38,46,47,48]. NB, although still effective, presents the lowest AUCs overall (0.80–0.83 in mpMRI and 0.695–0.80 in bpMRI) [37,48,49]. NN, DT, and LR models using bpMRI yield AUCs ranging from 0.71 to 0.936, depending on the study and configuration [38,43,48,50,51,52,53,54]. The literature review and the results of our study indicate the need to optimize the analysis process, regarding input variables and model choice and the need to standardize the pre-processing analysis steps.

Differentiating ciPCa from csPCa represents an initial critical step in PCa management. However, within the csPCa spectrum, accurate grading—especially the distinction between ISUP grades 1, 2, and ≥3—is essential because it significantly influences treatment strategies. [55] ISUP grade 1 (GS 6, 3 + 3) is often suitable for active surveillance, while ISUP grade 2 (GS 7, 3 + 4) may necessitate treatment for its limited aggressiveness, and ISUP grade ≥ 3 denotes more aggressive disease that warrants immediate intervention [56]. Accurate risk stratification is therefore essential to prevent both overtreatment of low-risk cases and under-management of potentially aggressive disease [57].

A significant disparity exists in PCa research concerned more with detection methods than with the grading and management of low-grade tumors. Twilt et al. observed that only a minority of studies employ ML for ISUP grade prediction using radiomic features. Algorithms’ efficiency to detect high-grade lesions ISUP ≥ 4 is usually high, while that to distinguish intermediate from low-grade lesions is not consistent across studies [58]. Indicatively, Abraham et al., applying a Convolution Neural Network (CNN) to T2W-, DWI-, and ADC-derived metrics, reported low AUC values, especially in low-grade lesions [AUC: 0.626 (GS 6~ISUP = 1), 0.535 (GS 3 + 4~ISUP = 2), 0.379 (GS 4 + 3~ISUP = 3), 0.761 (GS 8~ISUP = 4), and 0.847 (GS ≥ 9~ISUP = 5)] [59]. Low efficiencies were also reported by McGarry et al., who combined four MRI contrasts (T2W, ADC 0–1000, ADC 50–2000, and DCE) to generate Gleason probability maps, achieving low AUC (0.56) for distinguishing GS 4–5 from GS 3, but higher performance (AUC = 0.79) for benign vs. malignant classification [60]. Improved performance was reported by Chaddad et al. in two different studies, where they used two different methodological approaches to lesion grading. At first, they used Joint Intensity Matrix and Gray-Level Co-Occurrence Matrix features from The Cancer Imaging Archive (TCIA) dataset and reported lower than their expectation AUC values of 78.4% (GS ≤ 6), 82.35% (GS 3 + 4), and 64.76% (GS ≥ 4 + 3), which they attribute to omission of key clinical and morphological features [61]. Later, they applied an RF classifier with zone-based features achieving better AUC value in low-grade lesions and high-grade lesions (GS 6 AUC = 0.83 and GS ≥ 4 + 3 AUC = 0.77, respectively), while AUC was importantly decreased in intermediate lesions of GS 3 + 4 (AUC = 0.73). Similar performance was reported by Nketiah et al., who used logistic regression on texture features from T2W, ADC, and DCE, [AUCs of 0.83 Angular Second Moment (ASM) for GS 3 + 4 vs. 4 + 3] [62]. Higher AUC values were achieved by Jensen et al., who used a kNN model in which they were introduced T2WI- and DWI-derived features, highlighting the effect of lesion location, since AUC values were 0.96 in PZ and 0.83 in TZ to identify ISUP 1 or 2, 0.98 in PZ and 0.94 in TZ for ISUP 3, and 0.91 in PZ and 0.87 in TZ for ISUP ≥ 4 [63]. Also, high performance was published by Fehr et al., who employed a Recursive Feature Selection–Support Vector Machine (RFS-SVM) with Synthetic Minority Oversampling Technique (SMOTE), achieving AUCs of 0.93 (GS 6 vs. ≥7) and 0.92 (GS 3 + 4 vs. 4 + 3), including both TZ and PZ lesions [64].

Our results are comparable to those reported in the literature when only radiology-derived features are used, but significantly higher when PSA values are included. Therefore, all these findings highlight considerable variability in ML-based ISUP grading. Standardized radiomic workflows, larger multicenter datasets, and prospective validation are critical to improving model reliability and clinical integration.

This study has several strengths, which mainly concern the methodology used. We tried to deploy a high-performance model, including the optimal combination of input parameters and discovering the most effective algorithm. Also, models’ generality was improved, including imaging data from four different MRI systems, of which acquisition protocols were not standardized and tested, applying both cross-validation and held-out tests. However, our study has several limitations. First, the held-out test was only performed in the model that differentiated benign from malignant prostate lesions regardless of their location. Second, the relatively small sample size may affect the robustness of our findings. Third, the study lacks an assessment of the impact of conventional radiological parameters such as prostate volume and does not incorporate other clinical variables or patient history data. Finally, features related to lesion perfusion were not extracted, as the imaging protocol did not include DCE sequences.

## 5. Conclusions

There is evidence that ML methods and radiomic analyses provide an objective evaluation of bpMRI data, contributing to PCa diagnosis and prognosis and avoiding invasive methods. Also, optimizing the methodology concerning the input variables and the used algorithm contributes to increasing the models’ performance. Therefore, there is need of multicenter studies including larger datasets to validate their efficiency in grading the lesions.

## Figures and Tables

**Table 1:** Clinical and epidemiological characteristics of patient cohort.

Variable	
No. of patients	214
Benign	113
Malignant	101
Median age (y) (mean ± std)	66.00 ± 7.77
Median PSA level (ng/mL) (mean ± std)	8.06 ± 7.03
Median prostate volume (mm^3^) (mean ± std)	61.17 ± 40.28
Histopathologically confirmed lesions	214
Gleason score (GS)	
GS < 6	62
GS = 6	51
GS = 7 (3 + 4)	46
GS = 7 (4 + 3)	37
GS > 7	18
International Society of Urological Pathology (ISUP) group	
ISUP = 1	113
SUP = 2	46
ISUP = 3	37
ISUP = 4	11
ISUP = 5	7
Lesion location	
Peripheral zone	158
Other zones	56

**Table 2:** Area under curve (AUC) results across different datasets and algorithms, evaluated under various discrimination criteria, for both the entire prostate gland and the peripheral zone.

		Entire Prostate	Peripheral Zone	
	Discriminate Criterion	GS* ≤ 6 vs. GS > 6	ISUP* 2 vs. ISUP 3	ISUP* 1 vs. ISUP 2&3
**Algorithm Models**	Features	AUC	AUC	AUC
**Random Forest**	T2W*	0.747	0.670	0.739
	DWI*	0.711	0.762	0.670
	T2W + DWI	0.735	0.709	0.603
	T2W + DWI + PSA*	0.946	0.995	0.995
**kNN**	T2W	0.738	0.545	0.756
	DWI	0.721	0.713	0.606
	T2W + DWI	0.726	0.632	0.599
	T2W + DWI + PSA	0.868	0.784	0.898
**Naive Bayes**	T2W	0.763	0.726	0.746
	DWI	0.728	0.686	0.688
	T2W + DWI	0.786	0.725	0.675
	T2W + DWI + PSA	0.830	0.921	0.975
**Logistic Regression**	T2W	0.755	0.736	0.746
	DWI	0.719	0.710	0.622
	T2W + DWI	0.807	0.738	0.616
	T2W + DWI + PSA	0.884	0.972	1.000
**SVM**	T2W	0.717	0.369	0.750
	DWI	0.703	0.845	0.545
	T2W + DWI	0.736	0.715	0.647
	T2W + DWI + PSA	0.957	1.000	1.000
**Decision Tree**	T2W	0.721	0.582	0.747
	DWI	0.630	0.634	0.553
	T2W + DWI	0.678	0.609	0.678
	T2W + DWI + PSA	0.953	0.962	1.000
**Neural Network**	T2W	0.753	0.597	0.760
	DWI	0.703	0.824	0.569
	T2W + DWI	0.726	0.769	0.651
	T2W + DWI + PSA	0.992	0.989	0.989

*- GS*, Gleason score; ISUP*, International Society of Urological Pathology; T2W*, T2-weighted; DWI*, diffusion-weighted; PSA*, Prostate-Specific Antigen.

**Table 3:** *p*-values from Pairwise DeLong test, which assesses whether the differences in (area under curve) AUC between pairs of models are statistically significant for different classification tasks.

Delong *t*-Test, *p*-Values (GS* ≤ 6 vs. GS* > 6)							
Model 1/Model 2	NB*	kNN*	LR*	SVM*	DT*	RF*	NN*
NB*	-	0.6231	0.0144	0.0000	0.0188	0.0003	0.0005
kNN	0.0623	-	0.0392	0.0000	0.0311	0.0003	0.0005
LR*	0.0014	0.0392	-	0.0005	0.7591	0.1472	0.0018
SVM*	0.0000	0.0000	0.0005	-	0.0023	0.0332	0.0005
DT*	0.0019	0.0311	0.7591	0.0023	-	0.3381	0.0321
RF*	0.0000	0.0003	0.1472	0.0332	0.3381	-	0.0045
NN*	0.0005	0.0018	0.0005	0.0005	0.0321	0.0045	-
Delong *t*-test, *p*-values (ISUP* 2 vs. ISUP* 3)							
Model 1/Model 2	NB*	kNN	LR*	SVM*	DT*	RF*	NN*
NB*	-	0.0219	0.0501	0.0194	0.0426	0.0082	0.0445
kNN	0.0219	-	0.1028	0.0021	0.1221	0.0155	0.4658
LR*	0.0501	0.1028	-	0.0652	0.9284	0.5712	0.0489
SVM*	0.0194	0.0021	0.0652	-	0.0781	0.1449	0.0187
DT*	0.0426	0.1221	0.9284	0.0781	-	0.6358	0.0018
RF*	0.0082	0.0155	0.5712	0.1449	0.6358	-	0.0189
NN*	0.0445	0.4658	0.0489	0.0187	0.0018	0.0189	-
Delong *t*- tets, *p*-values (ISUP* 1 vs. ISUP* 2,3)							
Model 1/Model 2	NB*	kNN*	LR*	SVM*	DT*	RF*	NN*
NB*	-	0.5420	0.0368	0.0012	0.0031	0.0058	0.0248
kNN	0.5420	-	0.0793	0.0007	0.0020	0.0045	0.0048
LR*	0.0368	0.0793	-	0.0180	0.0736	0.1199	0.0112
SVM*	0.0012	0.0007	0.0180	-	0.0643	0.1161	0.0187
DT*	0.0031	0.0020	0.0736	0.0643	-	0.8378	0.0287
RF*	0.0058	0.0045	0.1199	0.1161	0.8378	-	0.0385
NN*	0.0248	0.0048	0.0112	0.0187	0.0287	0.0385	-

*.NB*, Naïve Bayes; kNN*, k-Neural Network; LR*, logistic regression; SVM*, Support Vector Machine; *DT, Decision Tree; RF*, Random Forest, *NN: Neural Network; GS*, Gleason score; ISUP*, International Society of Urological Pathology.

**Table 4:** Results of held-out cross validation by using datasets 1, 2, and 3 for training and dataset 4 as the test set, incorporating T2W, DWI, and PSA as input features. The classification task was based on a Gleason score discrimination cut-off of 6, distinguishing benign (GS ≤ 6) from malignant (GS ≥ 7) prostate lesions.

Algorithm	Random Forest	kNN	Naïve Baiyes	Logistic Regression	SVM	Decision Tree	Neural Network
Evaluation method	Area under curves (AUC) values						
Cross-validation	0.946	0.868	0.830	0.884	0.957	0.953	0.992
Held-out set	0.814	0.764	0.700	0.764	1.000	0.929	0.936

**Figure A1:**
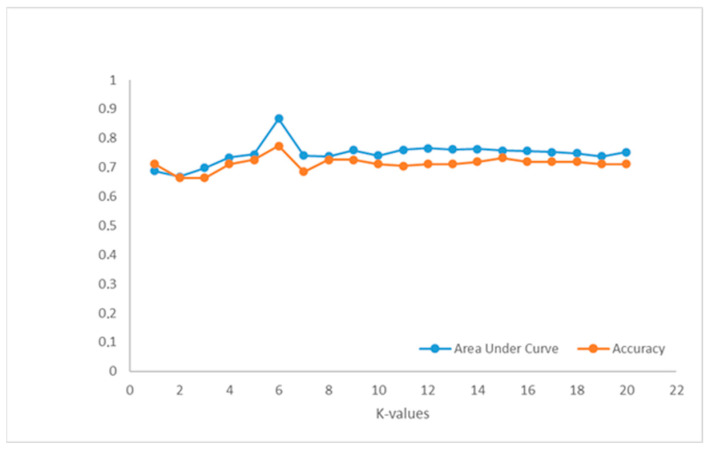
The selection of k value for optimization of Neural Network (kNN) algorithm with empirical performance with plot of area under curve and accuracy across k-values range from 0 to 20.

## Data Availability

Data is unavailable due to privacy or ethical restrictions.

## Abbreviations

The following abbreviations are used in this manuscript:

PCa	Prostate Cancer
PSA	Prostate-Specific Antigen
DRE	Digital Rectal Examination
EAU	European Association of Urology
ESTRO	European Society for Radiotherapy & Oncology
SIOG	International Society of Geriatric Oncology
cs-PCa	Clinically Significant Prostate Cancer
mpMRI	Multiparametric Magnetic Resonance Imaging
T2W	T2-Weighted Imaging
DWI	Diffusion-Weighted Imaging
DCE	Dynamic Contrast-Enhanced Sequences
AUC	Area Under the Receiver Operating Characteristic Curve
PI-RADS v2.1	Prostate Imaging Reporting and the Data System Version 2.1
MRI	Magnetic Resonance Imaging
ML	Machine Learning
PZ	Peripheral Zone
TZ	Transition Zone
TRUS	Τransrectal Ultrasound Guided
FBW	Fixed Bin-Width
IBSI	Imaging Biomarkers Standardization Initiative
GLCM	Gray-Level Co-Occurrence Matrix
GLSZM	Gray-Level Size Zone Matrix
GLRLM	Gray-Level Run Length Matrix
GLDM	Gray-Level Dependence Matrix
k-NN	K-Nearest Neighbors
NB	Naive Bayes
LR	Logistic Regression
SVM	Support Vector Machine
DT	Decision Tree
RF	Random Forest
NN	Neural Network
LASSO	Least Absolute Shrinkage and Selection Operator
C	Cost
ε	Regression Loss Epsilon
ReLU	Rectified Linear Unit
ROC	Receiver Operating Characteristic
GS	Gleason Score
ISUP	International Society of Urological Pathology
TNM	Tumor Nodule Metastasis
ciPCa	Clinical Insignificant Prostate Cancer
ADC	Apparent Diffusion Coefficient
CL	Clinical Feature
CNN	Convolutional Neural Network
TCIA	The Cancer Imaging Archive
ASM	Angular Second Moment
RFS-SVM	Recursive Feature Selection–Support Vector Machine
SMOTE	Synthetic Minority Oversampling Technique

## Appendix A

Radiomic features were extracted from the pre-processed T2W and DWI images using the Pyradiomics v1.3.0, following the Imaging Biomarkers Standardization Initiative (IBSI) processing protocol.

**Table A1:** The extracted features of T2-weighted and diffusion-weighted images.

Classes of Features	Features
First Order Statistics	Energy
	Total Energy
	Entropy
	Minimum
	10 Percentile
	90 Percentile
	Mean
	Median
	Maximum
	Interquartile Range
	Range
	Mean Absolute Deviation
	Robust Mean Absolute Deviation
	Root Mean Squared
	Skewness
	Kurtosis
	Uniformity
	Variance
Shape-Based (3D)	Mesh Volume
	Voxel Volume
	Surface Area
	Surface Volume Ratio
	Sphericity
	Maximum 2D Diameter Column
	Maximum 2D Diameter Row
	Maximum 2D Diameter Slice
	Maximum 3D Diameter
	Major Axis Length
	Minor Axis Length
	Least Axis Length
	Elongation
	Flatness
Gray-Level Co-Occurrence Matrix (GLCM)	Autocorrelation
	Joint Average
	Cluster Prominence
	Cluster Shade
	Cluster Tendency
	Contrast
	Correlation
	Difference Average
	Difference Entropy
	Difference Variance
	Joint Energy
	Joint Entropy
	Informational Measure of Correlation (Imc1)
	Informational Measure of Correlation (Imc2)
	Inverse Difference Moment (Idm)
	Inverse Difference Moment Normalized (Idmn)
	Inverse Difference (Id)
	Inverse Difference Normalized (Idn)
	Inverse Variance
	Maximum Probability
	Sum Entropy
	Sum Squares
Gray-Level Size Zone Matrix (GLSZM)	Small Area Emphasis
	Large Area Emphasis
	Gray-Level Non-Uniformity
	Gray-Level Non-Uniformity Normalized
	Size Zone Non-Uniformity
	Size Zone Non-Uniformity Normalized
	Zone Percentage
	Gray-Level Variance
	Zone Entropy
	Zone Variance
	Low Gray-Level Run Emphasis
	High Gray-Level Run Emphasis
	Small Area High Gray-Level Emphasis
	Small Area Low Gray-Level Emphasis
	Gray-Level Variance
	Large Area High Gray-Level Emphasis
	Large Area Low Gray-Level Emphasis
	High Gray-Level Zone Emphasis
	Low Gray-Level Zone Emphasis
Gray-Level Run Length Matrix (GLRLM)	Short Run Emphasis
	Long Run Emphasis
	Gray-Level Non-Uniformity
	Gray-Level Non-Uniformity Normalized
	Run Length Non-Uniformity
	Run Length Non-Uniformity Normalized
	Run Percentage
	Gray-Level Variance
	Run Variance
	Run Entropy
	Long Run High Gray-Level Emphasis
	Long Run Low Gray-Level Emphasis
	Short Run High Gray-Level Emphasis
	Short Run Low Gray-Level Emphasis
Gray-Level Dependence Matrix (GLDM)	Large Dependence Emphasis
	Small Dependence Emphasis
	Gray-Level Non-Uniformity
	Dependence Non-Uniformity
	Dependence Non-Uniformity Normalized
	Dependence Variance
	Dependence Entropy
	High Gray-Level Emphasis
	Large Dependence High Gray-Level Emphasis
	Large Dependence Low Gray-Level Emphasis
	Low Gray-Level Emphasis
	Small Dependence Emphasis
	Small Dependence High Gray-Level Emphasis
	Small Dependence Low Gray-Level Emphasis

## Appendix B

The selection of k value on kNN algorithm based on empirical performance, through cross-validation, tested different values of k (e.g., 1 to 20) and found this gives the best accuracy and AUC value or lowest error on your validation set. Also, the selection of even k should be used to avoid ties in classification decisions. Error parameters are not supported by Orange Data Mining Software.

Empirical performance of k selection in this study justified in Table A2 and illustrated in Figure A1.

**Table A2:** Area under curve and accuracy for different k values from 0 to 20.

k	Area Under Curve (AUC)	Accuracy
1	0.688	0.712
2	0.668	0.664
3	0.699	0.664
4	0.734	0.712
5	0.745	0.726
6	0.868	0.774
7	0.74	0.685
8	0.738	0.726
9	0.759	0.726
10	0.741	0.712
11	0.761	0.705
12	0.766	0.712
13	0.762	0.712
14	0.763	0.719
15	0.758	0.733
16	0.756	0.719
17	0.753	0.719
18	0.749	0.719
19	0.738	0.712
20	0.752	0.712
